# Ewing’s sarcoma of the ilium mimicking inflammatory arthritis of the hip: a case report

**DOI:** 10.4076/1757-1626-2-6487

**Published:** 2009-06-29

**Authors:** Manzoor Ahmed Halwai, Bashir Ahmed Mir, Mubashir Maqbool Wani, Arshad Bashir, Anwar Hussain

**Affiliations:** Department of Orthopaedics, Government Hospital for Bone and Joint Surgery BarzullahSrinagar, 190005India

## Abstract

**Introduction:**

Ewing’s sarcoma is second most common primary malignant bone neoplasm in children and adolescents. We report a case of Ewing’s sarcoma of ilium in an 18-year-old female mimicking clinically and radiologically inflammatory arthritis of hip joint, a rare entity not mentioned in literature.

**Case presentation:**

An 18-year-old girl presented with pain hip, limp and fever off and on. Patient had restricted range of motion, raised erythrocyte sedimentation rate, leucocytosis and radiograph showed reduced joint space. Magnetic resonance imaging pelvis revealed an altered marrow signal of acetabulum with a large soft tissue component. Histopathology revealed a malignant round cell tumor consistent with Ewing’s sarcoma.

**Conclusion:**

Classical clinical and radiological presentation of Ewing’s sarcoma of ilium may not be the rule. One should be highly suspicious of the disease even if there is no direct pointer to the disease as was encountered in our case.

## Introduction

Ewing’s sarcoma is a highly malignant tumor of bone and soft tissue in children and adolescents. It was named after James Ewing, who first described it as a distinct entity [1]. ES is the second most common primary malignant tumor of bone in children with a characteristic predilection for an age group between 10-20 years [2]. It is rare in black populations of America or Africa and in children of Asian origin [3]. Etiology is unknown. However, in 85% of ES, the pathognomonic t(11:22) chromosomal translocation is found. The location of ES is most often in the pelvis and lower extremity [4]. In 12.5% cases of ES, iliac bone is the site of origin. Clinically, the most common presenting symptom is pain (90%), followed by swelling (70%) [5]. Pain may be present for months and years before patient seeks medical attention [6]. Rectal and urinary complaints may result when the neoplasm is located on the innominate bone. In ES fever, weight loss, secondary anemia, leucocytosis, and an increase in the sedimentation rate are not frequently seen unless the case is quite advanced. These may lead to confusion with osteomyelitis and lymphoma. Radiologically, it was classically described as a central, diaphyseal, lytic tumor, which is often permeative and has a lamellated or ‘onion skin’ periosteal reaction affecting a long bone, and associated with soft-tissue mass. The bone lesions are usually lytic, but may be sclerotic or mixed. Most of the lesions are diaphyseal or metadiaphyseal. ES may be misdiagnosed as benign disorders. Cases of ES of the ilium mimicking sacroiliitis have been reported [7,8]. ES of the tibia may mimic osteomyelitis. ES may present as monoarthritis knee [9], spondylolisthesis and as postpartum back pain. ES ilium mimicking inflammatory arthritis hip has not been reported in the literature to the best of our knowledge.

## Case presentation

**Figure 1.**
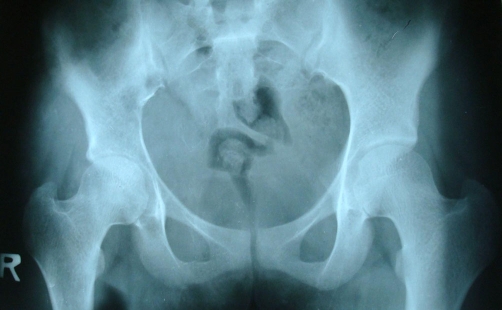
Plain X-ray pelvis AP view showing reduced right hip joint space with hard to pick changes in the iliac bone.

**Figure 2.**
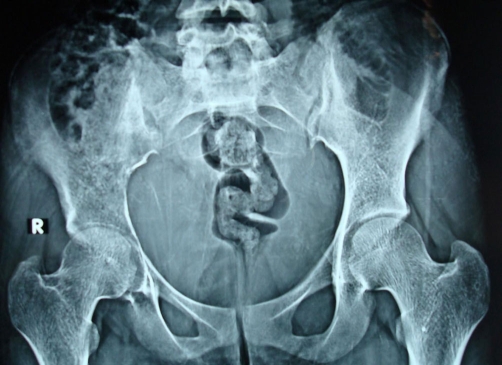
Digital X-ray pelvis AP view showing reduced right hip joint space with displaced fat planes suggestive of effusion.

**Figure 3.**
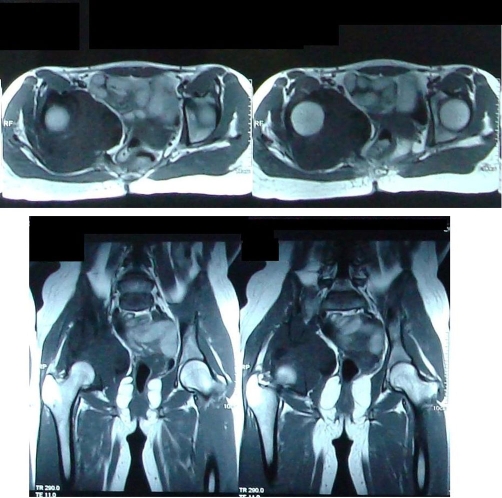
MRI pelvis of the same patient suggestive of mass lesion of right acetabulum.

**Figure 4.**
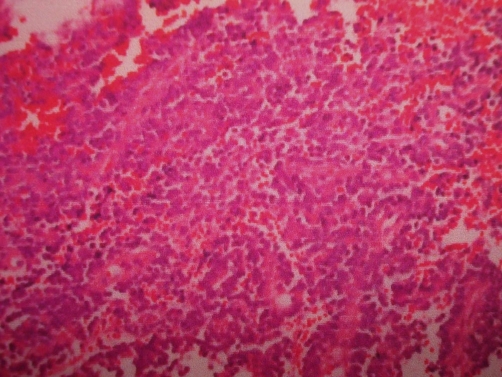
Histopathology of the patient’s biopsy showing densely packed small round cells consistent with ES.

We report a case of 18-year-old female patient, Asian Indian in origin presenting with one-month history of pain right hip, limp and fever off and on. On examination patient was febrile, there was localized tenderness over ilium, no swelling, had restricted internal and external rotation of right hip and an apparent lengthening of 1.5 cm. Patient had no rectal or urinary complaints. Investigations revealed a sedimentation rate of 45 mm, hemoglobin 10.6 gm%, TLC of 12500, PCR for tuberculosis was negative. Plain radiograph pelvis in AP view showed reduced right hip joint space with subtle, hard to pick changes in the right ilium (Figure 1). Digital radiograph pelvis in same view showed reduced right hip joint space with displaced fat planes suggestive of possible effusion. Left hip was normal (Figure 2). Patient was treated with conventional non steroidal anti-inflammatory drugs, intravenous antibiotics and a below knee skin traction with little benefit to her. MRI pelvis was done which revealed an altered marrow signal of right acetabulum and iliac bone (hypointense on T1W and hyperintense on T2W/STIR images) with a large soft tissue component on its posterior-medial aspect indenting the urinary bladder. The adjacent muscles and intramuscular planes were normal. Both SI joints were normal. The left hip joint was normal. There was no pelvic lymphadenopathy or free fluid (Figure 3). Keeping in view the MRI findings, biopsy was sought. The incision was given over the suspected area and tumor tissue was identified. This tissue was preserved for histopathological examination and sent to the pathologist. The pathologist reported it as densely packed, small, round cells arranged into sheets or lobules separated by fibrovascular connective tissue septa consistent with ES (Figure 4). Immunohistochemistry for CD99 marker was positive. The patient is being treated currently with multidrug chemotherapy using drugs, which include vincristine, doxorubicin, ifosfamide and etoposide. Patient has now been on primary chemotherapy for 5 months and it will be continued for a month more.

## Discussion

Ewing’s sarcoma accounts for approximately 10% of malignant bone tumors. The male: female ratio is 3:2. Clinically presents as pain with swelling and a palpable mass. Back pain and parathesias can be associated with paraspinal or pelvic tumors. Common nonspecific systemic manifestations include fever, fatigue, weight loss, and anemia mimicking acute or chronic infection. Additional symptoms and signs depend on the specific location of the tumor. Ewing’s sarcoma can affect all bones, but is slightly more common in tubular bones. The flat bones most often involved are the ilium, ischium, rib, and scapula. More than 50% of patients have a soft tissue component. Ewing’s sarcoma is one of the few bone sarcomas that affect the bones of the hands and feet, although in less than 5% of patients. Hematogenous dissemination is frequent to the lung, bones, and bone marrow. Extraosseous extension is common. On plain radiographs classic appearance is an intramedullary lesion with aggressive permeative or moth-eaten bone destruction. Periosteal new bone can be laminated or interrupted with a “sunburst” appearance. A Codman’s triangle is less commonly seen. A characteristic, but not specific, appearance is subperiosteal extension with subperiosteal bone destruction producing a concave “saucerization” defect.

After going through literature on ES of ilium and surfing through pubmed data base, no case of ES of the ilium mimicking inflammatory arthritis hip has been reported to the best of our knowledge. Although ES ilium mimicking sacroiliitis have been reported [7,8]. The radiologic appearance of inflammatory and tumorous lesions in the iliac bone is characterized by destructive alterations and consolidations simultaneously. This pattern is nonspecific. The value of plain films of this area is compromised by the anatomy of the iliac bone and by overlying structures. Therefore MRI, computer tomography and bone scans are necessary [10]. CT is helpful in defining the tumor of ES by showing the pattern of bone destruction and the associated soft-tissue mass. MRI is more sensitive than CT in showing soft-tissue involvement and bone marrow metastases. Definitive diagnosis is by an open biopsy and histological examination combined with immunochemistry and cytogenetics.

Before starting treatment patients should be evaluated by specialists from all disciplines (e.g., radiologist, chemotherapist, pathologist, surgeon or orthopedic oncologist, and radiation oncologist) as early as possible. The surgeon or orthopedic oncologist who will perform the definitive surgery should be involved prior to or during the biopsy so that the incision can be placed in an acceptable location. The successful treatment of patients with Ewing family of tumors (EFT) requires systemic chemotherapy in conjunction with either surgery or radiation therapy or both modalities for local tumor control. In general, patients receive preoperative chemotherapy prior to instituting local control measures. Multidrug chemotherapy for EFT always includes vincristine, doxorubicin, ifosfamide, and etoposide. Most protocols use cyclophosphamide as well. We did not use cyclophosphamide in our patient. Duration of primary chemotherapy ranges from 6 months to approximately 1 year. While surgery is effective and appropriate for patients who can undergo complete resection with acceptable morbidity, children who have unresectable tumors or who would suffer loss of function are treated with radiation therapy alone. Those who undergo gross resections with microscopic residual disease may benefit from adjuvant radiation therapy. Prognosis depends on extent of the disease, size and location of the tumor, presence or absence of the tumor metastasis, tumor response to therapy, age,and disease relapse. Most centers today report long term survival of 60% to 70%. The worst prognostic factor is the presence of distant metastasis. Even with aggressive treatment, patients with metastasis have only a chance of 20% long term survival. Histological grades are of no prognostic significance. As already mentioned fever, anemia and elevation of WBC, ESR and lactate dehydrogenase have been reported to indicate more extensive disease and a worse prognosis.

## Conclusion

The classical clinical and radiological presentation of ES of iliac bone may not be the rule; one should be highly suspicious of the disease even if there is no direct pointer to the disease as was encountered in our case. Orthopaedicians, rheumatologists and radiologists should be alert to this rare atypical occurrence.

## Abbreviations

CT: Computed tomography
ES: Ewing’s sarcoma
ESR: Erythrocyte sedimentation rate
MRI: Magnetic resonance imaging
TLC: Total leucocyte count
